# Plasma levels of lysophosphatidic acid in ovarian cancer versus controls: a meta-analysis

**DOI:** 10.1186/s12944-015-0071-9

**Published:** 2015-07-15

**Authors:** Yi-Yang Li, Wen-Chao Zhang, Jia-Ling Zhang, Chang-Jun Zheng, He Zhu, Hui-Mei Yu, Li-Mei Fan

**Affiliations:** Department of Gynaecology, the First Hospital of Jilin University, Changchun, 130041 China; Center for Reproductive Medicine, Renji Hospital, Shanghai Jiao Tong University School of Medicine, Shanghai, China; Department of Gynaecology and Obstetrics, the Second Hospital of Jilin University, Ziqiang Street NO. 218, Nanguan District, Changchun, 130041 People’s Republic of China; Deparment of Pathology and Pathophysiology, School of Basic Medical Sciences, Changchun, 130021 China

**Keywords:** Lysophosphatidic acid, Ovarian cancer, Bioactive phospholipid, Meta-analysis

## Abstract

**Background:**

In this study, using a meta-analysis approach, we examined the correlation between serum levels of lysophosphastidic acid (LPA) and ovarian cancer (OC).

**Methods:**

Relevant published studies were identified from multiple scientific literature databases by using a pre-determined electronic and manual search strategy. The search results were screened through a multi-step process to select high-quality case–control studies suitable for the present meta-analysis. Mean values and standardized mean differences (SMD) were calculated for plasma LPA levels. Two investigators independently extracted the data from the studies and performed data analysis using STATA software version 12.0 (Stata Corp, College Station, TX, USA).

**Results:**

Nineteen case–control studies met our selection criteria and contained a total of 980 OC patients, 872 benign controls and 668 healthy controls. Our meta-analysis results revealed that the plasma levels of LPA in OC patients were significantly higher than benign controls (SMD = 2.36, 95 % CI: 1.61–3.10, *P* <0.001) and healthy controls (SMD = 2.32, 95 % CI: 1.77–2.87, *P* <0.001). Subgroup analysis by ethnicity showed that the plasma LPA levels in OC patients were significantly higher than the benign controls only in Asian populations (SMD = 2.52, 95 % CI: 1.79–3.25, *P* <0.001). However, a comparison between healthy controls and OC patients revealed that, in both Asians and Caucasians, the OC patients displayed significantly higher plasma LPA levels compared to healthy controls (all *P* <0.05).

**Conclusion:**

Our meta-analysis showed strong evidence that a significantly higher plasma LPA levels are present in OC patients, compared to benign controls and healthy controls, and plasma LPA levels may be used as a biomarker or target of OC.

## Introduction

Ovarian cancer (OC) is the deadliest among gynecological cancers, with 5-year survival rates ranging between 30-50 %. OC is not a single disease but is a collection of diverse tumors with distinct morphologies and genetic deficiencies. The pathogenesis of OC is not well characterized, but most OCs occurs spontaneously, with only 5-10 % of the cases linked to a genetic predisposition. According to the latest estimates, OC is the 7^th^ most common cancer worldwide, and the age-standardized incidence rates range from more than 11 per 100,000 women in central and eastern Europe to less than 5 per 100,000 in parts of Africa, and is the eighth most common cause of cancer death in women globally [1]. OC often has no overt symptoms at early stages and the disease is usually advanced at diagnosis, with metastatic spread, which is the main reason for the high death rates associated with OC [2]. The late diagnosis and advanced metastatic stage severely limits treatment options and severely impacts the quality of life in OC patients [3]. Although most OC patients with an advanced disease initially respond to first-line therapy, only 10-15 % will maintain a complete response. Therefore, discovery of underlying mechanisms and disease factors promoting tumor growth and metastasis is of urgent need for developing novel tools for OC diagnosis and treatment [4].

LPA is a small bioactive phospholipid present in ascetic fluid and blood of OC patients [4]. The G protein-coupled receptors of the endothelial differentiation gene (Edg) family are stimulated by LPA and LPA mediated signaling effects cell proliferation, invasion, smooth muscle cell contraction, platelet aggregation, cell migration, cell survival, wound healing and alteration in morphology and differentiation of cells [5–7]. LPA mediated pathways are prominently linked to tumor growth and metastasis in various cancers and thus significant efforts are underway to understand the precise role of LPA and design effective intervention strategies. LPA is converted from lysophospholipids in the serum and plasma, and from phosphatidic acid in platelets and cancer cells [8]. LPA production from lysophospholipids requires the action of phospholipase A1 (PLA1)/PLA2 plus lysophospholipase D (lysoPLD), while the production of LPA from phosphatidic acid requires phospholipase D (PLD) plus PLA1/PLA2 activities [8, 9]. Previous studies showed that ovarian tumor cells are a major source of LPA and autotaxin (ATX)/lysophospholipase D (PLD) pathway is the primary LPA producing pathway in ovarian tumor cells [10]. Consistent with this, plasma levels of LPA are strongly associated with presence of ovarian tumors and plasma levels of LPA are significantly higher in OC patients compared to benign ovarian lesions [4, 11]. Furthermore, previous studies showed that increased LPA levels are closely associated with the elevated expression levels of other prominent metastasis promoters critical for OC progression [12, 13]. Nevertheless, perhaps due to the complexity of the LPA pathway and the diversity of its receptors, several other studies reported results contradicting the links between LPA and ovarian cancer [14, 15]. Therefore, we performed a meta-analysis to closely examine this issue and obtain a correlation between plasma LPA level and OC.

## Materials and methods

### Literature search

The following computerized databases were searched electronically (last updated search in May 30^st^, 2014) for published studies reporting a correlation between plasma LPA levels and OC: China BioMedicine (CBM), Cochrane Library, PubMed and China National Knowledge Infrastructure (CNKI). The following keywords and search terms were used: (“lysophosphatidic acid” or “MOPA” or “LPA” or “1-oleoyl-lysophosphatidic acid” or “monooleylphosphatidate” or “1-O-oleyllysophosphatidic acid”) and (“ovarian neoplasms” or “ovary neoplasms” or “ovary cancers” or “ovarian cancer” or “cancer of ovary” or “ovarian carcinoma” or “ovarian adenocarcinoma” or “ovarian tumor” or “EOC”). The language of publication was not a restriction in our search criteria. Bibliographies of closely related studies were further examined manually to identify additional studies relevant to this meta-analysis.

### Study selection

The inclusion criteria for selection of published studies for this meta-analysis were: (1) OC patients must be confirmed by pathological diagnosis; (2) study design must be case–control studies reporting the correlation between plasma LPA levels and OC; (3) the plasma LPA levels and sample size must be supplied; (4) published studies with full text. If a 50 % identity in study subjects were identified between two extracted studies, only the study with the largest sample size was enrolled. The latest and most complete study was chosen from studies published by same authors.

### Data extraction

Two investigators independently extracted data from the selected studies, based on the pre-determined selection criteria, and any disagreements were resolved by discussion and reexamination. The following information was extracted: first author, publication date, country and ethnicity, study type and design, sample size, sex and age of subjects, detection method for plasma LPA levels, and plasma levels of LPA in OC patients, benign controls and healthy controls.

### Quality assessment

To assess the quality of the selected studies, the two investigators used the criteria outlined in the Critical Appraisal Skill Program (CASP) (http://www.casp-uk.net/#!casp-tools-checklists/c18f8). The CASP criteria for case–control studies include Section A (CASP01 ~ CASP07), Section B (CASP08 ~ CASP09) and Section C (CASP10 ~ CASP11): clear focus in the study (CASP01); appropriate research questions and pertinent answers to the questions (CASP02); propriety in the case enrollment (CASP03); propriety in the control selection (CASP04); accuracy in the measurement of exposure factors for the least bias (CASP05); controls with other important confounding factors (CASP06); completeness of research results (CASP07); precision research results (CASP08); reliability of research results (CASP09); applicability of research results to the local population (CASP10); coherence of research results to other available evidence (CASP11).

### Statistical analysis

The summary standard mean differences (SMDs) and their 95 % confidence interval (CI) were calculated and Z test was used to estimate the effect size. The SMDs for plasma LPA levels were aggregated utilizing STATA software, version 12.0 (Stata Corp, College Station, TX, USA) independently by two investigators. If heterogeneity was detected, random-effects model was employed for meta-analysis; otherwise fixed-effects model was adopted. Cochran’s Q-statistic was used to evaluate the heterogeneity across the enrolled studies, *P* <0.05 referring to statistical significance. *I*^*2*^ test was used to provide further evidence of heterogeneity, with 0 % as no heterogeneity and 100 % as maximal heterogeneity [16, 17]. If heterogeneity was detected, meta-regression and subgroup analysis based on ethnicity and detection methods were conducted to explore potential influencing factors. The influence of any single study on the overall results was verified by sensitivity analysis. To ensure the accuracy of the results, publication bias was evaluated by constructing a funnel plot, with the symmetry of the funnel plot as evidence of no publication bias and vice versa. Classic fail-safe N and Egger’s linear regression test was used for verifying the results displayed by the funnel plot [18].

## Results

### Included studies

**Fig. 1:**
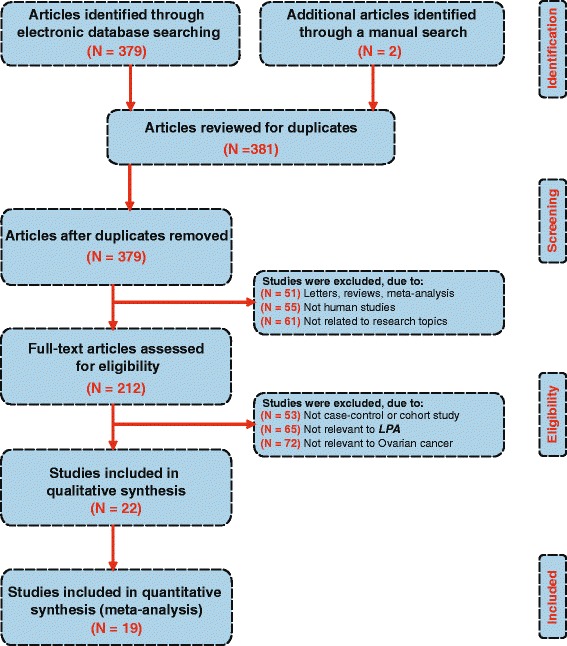
Flow chart shows study selection procedure. Nineteen studies were included in this meta-analysis

**Fig. 2:**
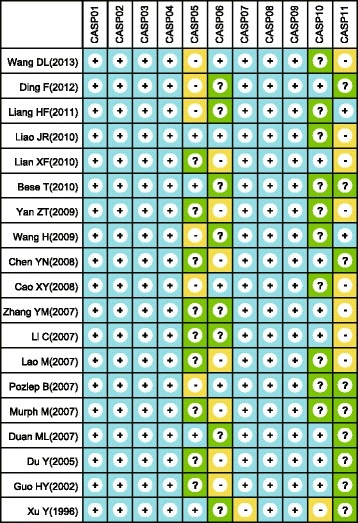
The methodological quality of eligible studies using critical appraisal skill program criteria (+: Yes; −: No; ? : Unclear)

Figure 1 presents the study inclusion process. A total of 381 studies were initially retrieved from database searches. From the retrieved studies, 169 studies were excluded for being duplicates (*n* = 2), letters, reviews or meta-analyses (*n* = 51), non-human studies (*n* = 55), or studies unrelated to the present topic (*n* = 61) and 190 were not for being non-case-control studies (*n* = 53), studies irrelevant to LPA (*n* = 65), or studies irrelevant to OC (*n* = 72), and another 3 others studies were removed because they failed to provide did not have the complete data. Finally, 19 case–control studies met the inclusion criteria [11–15, 19–32]. Fig. 2 presents the methodological quality assessment for these 19 studies. All 19 studies reported the correlation between plasma LPA levels and OC and were published between 1996 and 2013. Table 1 lists the demographic information on the OC patients and the baseline characteristics of the studies. Among the 19 studies, 16 studies were performed in Asian populations [China (*n* =15) and Turkey (*n* = 1)], and 3 were performed in Caucasians [Slovenia (*n* = 1) and USA (*n* = 2)]. Collectively, the 19 studies contained a total of 2,520 subjects, with 980 OC patients, 872 benign controls and 668 healthy controls. The plasma LPA levels in patients and controls were detected by ELISA (*n* = 13), bioassay (*n* = 4), Inorganic phosphorus (*n* = 1) and phosphorus determination (*n* = 1).

**Table 1:** Main characteristics and methodological quality of eligibly studies

First author	Year	Country	Ethnicity	Number			Age (years)			Methods
				Tumor	Benign	Normal	Tumor	Benign	Normal	
Wang DL [12]	2013	China	Asians	80	40	30	-	-	34 ~ 67	ELISA
Ding F [13]	2013	China	Asians	36	26	20	46.7	40.5	44.5	Bioassay
Liang HF [25]	2011	China	Asians	42	408	0	25 ~ 75			Bioassay
Liao JR-a [24]	2010	China	Asians	8	30	30	-	-	-	ELISA
Liao JR-b [24]	2010	China	Asians	40	30	30	-	-	-	ELISA
Lian XF [26]	2010	China	Asians	31	40	50	22 ~ 76	19 ~ 71	20 ~ 71	Inorganic phosphorus measurement
Bese T [11]	2010	Turkey	Asians	87	74	50	-	-	-	Bioassay
Yan ZT [21]	2009	China	Asians	76	35	29	-	-	-	ELISA
Wang H [23]	2009	China	Asians	30	32	36	28 ~ 68			ELISA
Chen YN [32]	2008	China	Asians	24	10	10	52	49	50	Bioassay
Cao XY [15]	2008	China	Asians	36	36	36	52.8	52.3	50.4	Phosphorus determination method
Zhang YM [20]	2007	China	Asians	50	44	20	29 ~ 73			ELISA
Li C-a [27]	2007	China	Asians	60	0	60	27 ~ 65			ELISA
Li C-b [27]	2007	China	Asians	60	0	60	27 ~ 65			ELISA
Lao M [28]	2007	China	Asians	80	40	40	19 ~ 68	19 ~ 65	18 ~ 55	ELISA
Pozlep B [19]	2007	Slovenia	Caucasians	142	0	78	48 ± 16.07		42.2	ELISA
Murph M [14]	2007	USA	Caucasians	26	27	25	-	-	-	ELISA
Duan ML-a [30]	2007	China	Asians	30	30	30	-	-	-	ELISA
Duan ML-a [30]	2007	China	Asians	30	30	30	-	-	-	ELISA
Du Y [31]	2005	China	Asians	37	0	30	53.5 ± 7.65		51.6 ± 6.57	ELISA
Guo HY-a [29]	2002	China	Asians	16	0	46	56.5 ± 8.64	-	-	ELISA
Guo HY-b [29]	2002	China	Asians	15	0	46	56.2 ± 10.9	-	-	ELISA
Xu Y [22]	1996	USA	Caucasians	34	0	48	-	-	-	ELISA

ELISA, enzyme linked immunosorbent assay

### Meta-analysis of findings

**Fig. 3:**
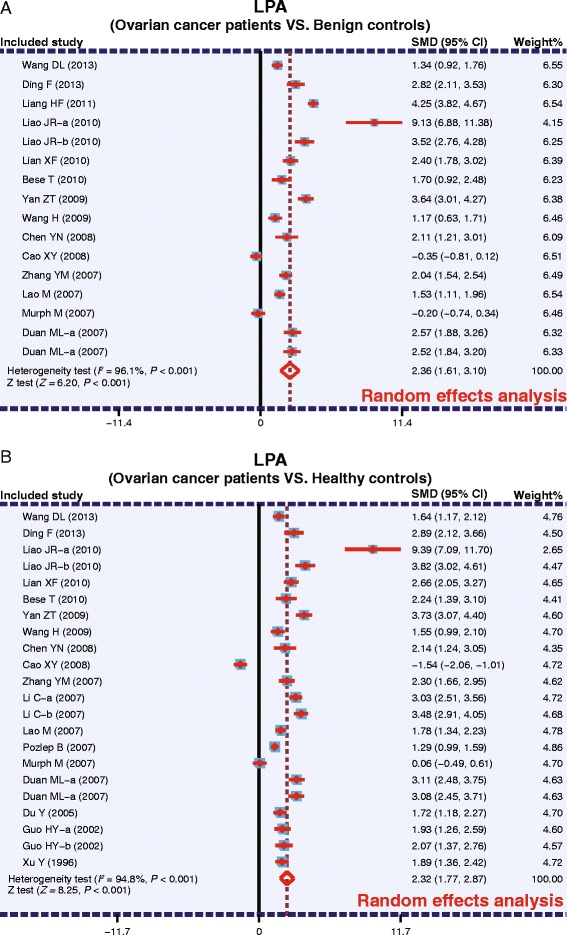
Forest plots for the relationship between plasma lysophosphatidic acid levels and ovarian cancer (**a**: Ovarian cancer patients VS. Benign controls; **b**: Ovarian cancer patients VS. Healthy controls)

**Fig. 4:**
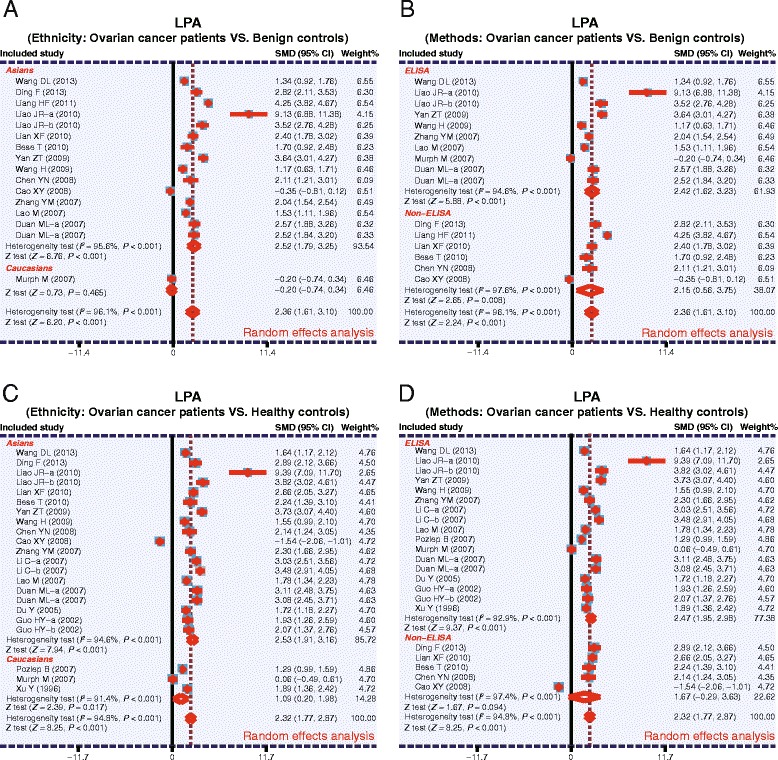
Subgroup analysis for the relationships between plasma lysophosphatidic acid levels and ovarian cancer (**a**: Ethnicity: Ovarian cancer patients VS. Benign controls; **b**: Methods: Ovarian cancer patients VS. Benign controls; **c**: Ethnicity: Ovarian cancer patients VS. Healthy controls; **d**: Methods: Ovarian cancer patients VS. Healthy controls)

As shown in Fig. 3, the pooled SMDs revealed that the plasma levels of LPA in OC patients were significantly higher than the benign controls (SMD = 2.36, 95 % CI: 1.61–3.10, *P* <0.001) and healthy controls (SMD = 2.32, 95 % CI: 1.77–2.87, *P* <0.001). The results of subgroup analysis based on ethnicity and detection methods are as follows: plasma LPA levels in OC patients are significantly higher compared to benign controls only among the Asian subjects (SMD = 2.52, 95 % CI: 1.79–3.25, *P* <0.001), as quantified by both ELISA (SMD = 2.42, 95 % CI: 1.62–3.23, *P* <0.001) and non-ELISA methods (SMD = 2.15, 95 % CI: 0.56–3.75, *P* = 0.008). Such a significant difference in plasma LPA levels between OC patients and benign controls was not found in the Caucasian population studied (*P* >0.05) (as shown in Fig. 4a-b). However, significantly higher plasma LPA levels were found in OC patients, compared to healthy controls, in both Asian (SMD = 2.53, 95 % CI: 1.91–3.16, *P* <0.001) and Caucasian populations (SMD = 1.09, 95 % CI: 0.20–1.98, *P* = 0.017) and in the ELISA subgroup (SMD = 2.47, 95 % CI: 1.95–2.98, *P* <0.001), but not in the non-ELISA subgroup (*P* >0.05) (Fig. 4c-d).

**Fig. 5:**
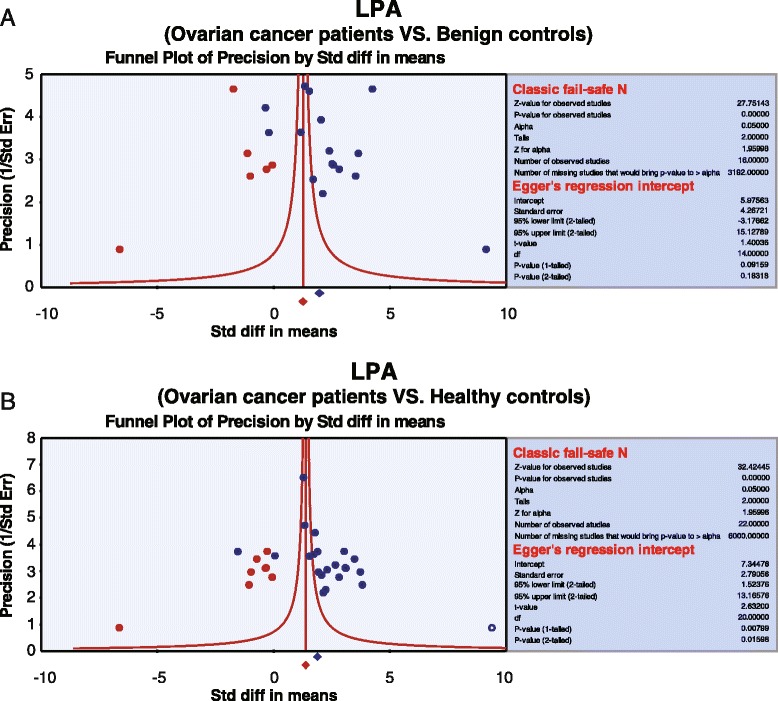
Funnel plot of publication biases on the relationship between plasma lysophosphatidic acid levels and ovarian cancer (**a**: Ovarian cancer patients VS. Benign controls; **b**: Ovarian cancer patients VS. Healthy controls)

Sensitivity analysis suggested that no single study had an impact on the overall statistical significance. For comparison between OC patients and benign controls, the constructed funnel plot was symmetrical, suggesting no publication bias, while it was asymmetrical for the comparison between OC patients and healthy controls (Fig. 5).

## Discussion

Based on the analysis presented in this study and the data from previous studies, LPA and OC appear to be strongly linked. For example, Hu et al. showed that LPA stimulates ovarian cancer cells by a dual mechanism: (1) LPA indirectly promotes tumor growth by acting as a chemoattractant to endothelial cells, resulting in increased angiogenesis and (2) directly increases the cell proliferation through cyclin D1 activation [33]. Autotaxin (ATX) is the predominant enzyme that produces LPA and ATX transgene expression in mouse mammary glands is sufficient to induce tumors. Conversely, ATX or LPA receptor knockdowns prevent bone metastasis and decrease tumor incidence and progression, respectively, in mouse models of chemically induced carcinogenesis. As such, ATX-LPA signaling in cancer is one of the prominent pathways for tumorigenesis and is a prime target for current therapeutic drug development [9]. Interestingly, contradictory results were obtained by several studies which could not find a clear correlation between LPA and OC. In order to examine this issue closer, we pooled the data from several high-quality published studies and performed a meta-analysis to obtain a better correlation between LPA and OC.

Our meta-analysis clearly showed that the plasma LPA levels in OC patients are significantly higher compared to the benign and healthy controls. Angiogenesis is a key factor for tumor growth and involves VEGF and the activation of VEGF receptors, Flt1 and KDR [19]. OC patients exhibit elevated serum levels of both VEGF and LPA [34]. ATX is an autocrine motility factor and a member of the ectonucleotide pyrophosphatase and phosphodiesterase family of enzymes, but also possesses lysophospholipase D activity. This enzymatic activity hydrolyzes lysophosphatidylcholine to generate the potent tumor growth factor and mitogen, LPA [8, 35]. ATX is highly overexpressed in OC, thus, tumor cells are the major source of LPA production in OC patients [8]. Notably, VEGF signaling was shown to further elevate ATX expression and, thus, OC patients have increased PLD activity and higher LPA plasma levels, compared to healthy controls.

A subgroup analysis was performed to identify factors influencing our study results. The potential factors tested were ethnicity, country and experimental method. Based on the limitations of the data available to us from the selected studies, we focused on ethnicity and detection methods for subgroup analysis. Our subgroup analysis based on ethnicity showed that OC patients from Asian populations exhibited higher plasma LPA levels compared to their benign and healthy counterparts, and the difference was statistically significant. Caucasian OC patients showed significantly higher plasma LPA levels compared to the healthy controls but not the benign counterparts. However, these results may be influenced by the fact that majority of the studies were performed in Asian populations (16 in Asians and 3 in Caucasians). Based on the detection methods, higher plasma LPA levels were observed in OC patients, compared to both benign and healthy controls, using ELISA based methods. The results from the analysis of non-ELISA based methods were largely in agreement with ELISA based results, but a significant correlation between LPA and OC was not observed between the OC patients and healthy controls using non-ELISA methods. This negative result may be due to the small sample size of non-ELISA methods. Collectively, our data provide strong evidence that LPA may be involved in OC development or is produced by the tumor and thus may be a tumor marker or target of treatment.

Our study has several significant limitations. Firstly, the dataset used in this study was limited by nineteen prospective trials, which might not be ideal for statistical analysis. Secondly, due to the heterogeneity of the disease, a uniform histopathological diagnosis would have ensured consistent diagnostic standards, which was not the case in the selected studies. Thirdly, some of the studies did not provide complete data and therefore plasma LPA levels at different stages of OC progression could not be compared. Fourthly, sample sizes of OC cases, controls and healthy controls varied widely. Therefore, for a more accurate statistical analysis, a comprehensive dataset and proportionate sample size may be needed. Future studies also must attempt to compare plasma LPA levels at different stages of OC progression to obtain more thorough disease correlations.

## Conclusion

In summary, a significantly higher plasma LPA levels were observed in OC patients compared to benign controls and healthy controls. Based on our results, we conclude that plasma LPA level is closely correlated to OC and may involve in the development of OC. However, further studies are needed to confirm our findings and explore therapeutic targets within the identified pathway.

## Abbreviations

OC: Ovarian carcinoma
LPA: Lysophosphatidic acid
Edg: Endothelial differentiation gene
NOS: Newcastle-Ottawa scale
SMDs: Standard mean differences
CI: Confidence interval
